# MRI-Based Surrogate Imaging Markers of Aggressiveness in Prostate Cancer: Development of a Machine Learning Model Based on Radiomic Features

**DOI:** 10.3390/diagnostics13172779

**Published:** 2023-08-28

**Authors:** Ignacio Dominguez, Odette Rios-Ibacache, Paola Caprile, Jose Gonzalez, Ignacio F. San Francisco, Cecilia Besa

**Affiliations:** 1 Department of Radiology, School of Medicine, Pontificia Universidad Católica de Chile, Santiago 8320000, Chile; jgdominguez@uc.cl; 2 Institute of Physics, Pontifical Catholic University of Chile, Av. Vicuña Mackenna 4860, Macul, Santiago 7820436, Chile; ovrios@uc.cl; 3 Medical Physics Unit, McGill University, Montreal, QC H4A 3J1, Canada; 4 Millennium Institute for Intelligent Healthcare Engineering, iHEALTH, ANID, Macul, Santiago 7820436, Chile; 5 School of Medicine, Pontifical Catholic University of Chile, Santiago 8320000, Chile; 6 Department of Urology, School of Medicine, Pontifical Catholic University of Chile, Santiago 8320000, Chile

**Keywords:** prostate cancer, Gleason score, texture analysis, bpMRI, machine learning, radiomics

## Abstract

This study aimed to develop a noninvasive Machine Learning (ML) model to identify clinically significant prostate cancer (csPCa) according to Gleason Score (GS) based on biparametric MRI (bpMRI) radiomic features and clinical information. Methods: This retrospective study included 86 adult Hispanic men (60 ± 8.2 years, median prostate-specific antigen density (PSA-D) 0.15 ng/mL^2^) with PCa who underwent prebiopsy 3T MRI followed by targeted MRI–ultrasound fusion and systematic biopsy. Two observers performed 2D segmentation of lesions in T2WI/ADC images. We classified csPCa (GS ≥ 7) vs. non-csPCa (GS = 6). Univariate statistical tests were performed for different parameters, including prostate volume (PV), PSA-D, PI-RADS, and radiomic features. Multivariate models were built using the automatic feature selection algorithm Recursive Feature Elimination (RFE) and different classifiers. A stratified split separated the train/test (80%) and validation (20%) sets. Results: Radiomic features derived from T2WI/ADC are associated with GS in patients with PCa. The best model found was multivariate, including image (T2WI/ADC) and clinical (PV and PSA-D) information. The validation area under the curve (AUC) was 0.80 for differentiating csPCa from non-csPCa, exhibiting better performance than PI-RADS (AUC: 0.71) and PSA-D (AUC: 0.78). Conclusion: Our multivariate ML model outperforms PI-RADS v2.1 and established clinical indicators like PSA-D in classifying csPCa accurately. This underscores MRI-derived radiomics’ (T2WI/ADC) potential as a robust biomarker for assessing PCa aggressiveness in Hispanic patients.

## 1. Introduction

Prostate cancer (PCa) is the most common noncutaneous malignancy and the second leading cause of cancer death in men in the United States, and its incidence is expected to double by the year 2030 [1]. PCa is a heterogeneous disease with various clinical and biological presentations [2]. Patients with PCa are classified according to their level of prostate-specific antigen (PSA), pathological evaluation (Gleason Score (GS)) [3], and clinical stage (i.e., T stage) [4], guiding treatment and prognosis. 

PCa risk patient stratification still constitutes a challenge due to the limitations of the current management algorithm. These include the low specificity of PSA and the overdetection of clinically nonsignificant low-grade neoplastic lesions by transrectal ultrasound (TRUS) guided biopsy, leading to overtreatment [5]. This has led to the search for new noninvasive methods that allow a better classification of these patients, including prebiopsy multiparametric magnetic resonance imaging (mpMRI), which has become an essential tool in PCa assessment [6,7], increasing the worldwide demand for prostate MRI. In clinical practice, evaluation of PCa on MRI is performed qualitatively by radiologists using the Prostate Imaging Data and Reporting System (PI-RADS v2.1) [8], which has shown an adequate diagnostic performance for predicting clinically significant PCa (csPCa) with areas under the curve (AUC) of 0.79–0.80 [9], with moderate inter-reader agreement (pooled k of 0.61, 95% CI 0.55–0.67) in a recent meta-analysis [10]. 

Extensive evidence supports the role of quantitative MRI-derived biomarkers in the risk stratification assessment of PCa patients. This strategy may improve diagnostic accuracy for imaging PCa diagnosis and reduce interobserver variability [11]. Among these, diffusion-weighted imaging (DWI) with derived apparent diffusion coefficient (ADC) maps [12,13] and T2-weighted imaging (T2WI) [14] have proven to be useful, showing a correlation with histopathology-based aggressiveness. Nevertheless, these quantitative MRI parameters’ discriminatory abilities are only moderate due to considerable overlaps between different GS, technical variations in image acquisition, and reproducibility, among other factors [15] limiting clinical application.

Quantitative analysis of MRI-derived parameters using radiomics and Machine Learning (ML) methods is a field of active research in PCa, with promising preliminary results for the detection of clinically significant PCa and the potential to overcome the limitations mentioned above, improving the MRI-driven diagnostic pathway [16,17]. Recent meta-analyses and systematic reviews have reported adequate performance and accuracy for PCa diagnosis and classification [18,19]. Furthermore, previous studies have indicated a significant correlation between T2WI/ADC textural features and GS [20,21].

PCa radiomics is a continuously evolving field of research with a high potential to offer noninvasive personalized biomarkers for risk stratification. This study aims to develop a noninvasive ML model to identify csPCa according to GS classification based on bpMRI radiomic features (T2WI/ADC) combined with clinical information (PSA, PSA-D, prostate volume) in a cohort of Hispanic patients, a population usually underrepresented in previously reported data. The study focuses on classifying csPCa (GS ≥ 7) vs. non-csPCa (GS = 6).

## 2. Materials and Methods

### 2.1. Patients 

**Figure 1:**
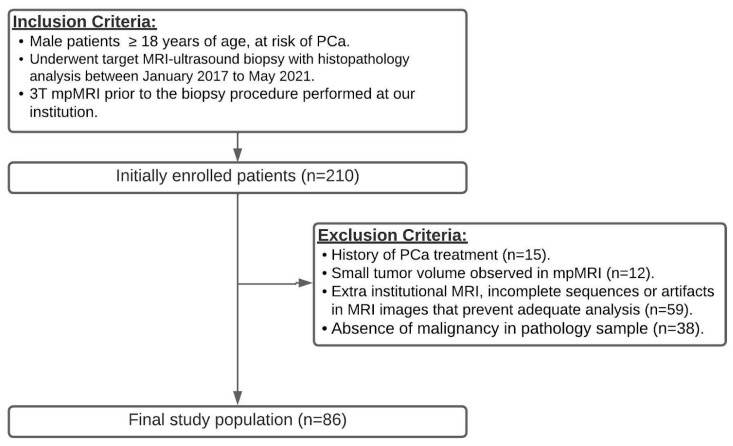
Flowchart of the patient population.

This single-center retrospective study was HIPAA compliant. Our local institutional ethical review board approved this study and waived the requirement for informed consent. Our urology database was queried to identify adult male patients with clinical suspicion of PCa who underwent a transrectal prostate biopsy with fusion technique (target MRI–ultrasound biopsy) between January 2017 and May 2021. All patients had a clinical indication for prostate biopsy based on elevated PSA levels or suspicious clinical examination. The search yielded 210 consecutive patients. The inclusion criteria were as follows: (a) patients aged ≥18 years at risk of PCa and (b) 3T multiparametric MRI (mpMRI) conducted before the biopsy procedure at our institution. Exclusion criteria comprised (a) a history of PCa treatment (surgery, radiotherapy, or hormonal therapy); (b) small tumor volume observed in bpMRI (maximum diameter ≤ 5 mm); (c) MRI performed outside the institution, incomplete sequences, or MRI images with artifacts that hindered adequate analysis; and (d) absence of malignancy in the pathology sample, as illustrated in Figure 1. Demographic data, biochemical tests, and pathology results were extracted from the clinical data system.

### 2.2. MR Imaging

A standard-of-care prostate mpMRI protocol at 3T (Philips Ingenia Gyroscan, Best, Netherlands) was acquired before biopsy according to PI-RADS v2.1 guidelines [8] using the standard 16-multichannel body coil and integrated spine-phased-array coil, as summarized in Table 1. Per protocol, patients were instructed to fast for 6 h before the MRI exam (for DCE-MRI purposes), avoid ejaculatory activity for at least 72 h before the examination, and perform bowel preparation before the MRI exam (administration of a fleet enema 4 h before testing). mpMRI included the following sequences: 3 plane T2WI, axial T1WI, axial DWI (b-values of 50, 500, and 1000 s/mm^2^), a separate acquisition of axial DWI (b-value of 2000 s/mm^2^), wide field of view (FOV) T1WI in phase and out phase images, and DCE imaging post administration of gadolinium contrast agent. All sequences had a slice thickness of 3 mm, with no gap. No endorectal coil was used at our institution. Table time acquisition time was approximately 30–40 min for this protocol.

### 2.3. Target MRI–Ultrasound Biopsy 

All selected patients underwent prebiopsy mpMRI to identify regions of PCa suspicion, followed by targeted MRI–ultrasound fusion and concurrent standard systematic TRUS-guided biopsy. Before the procedure, the prebiopsy mpMRI was interpreted by a dedicated clinical radiologist, and the location of a suspicious tumoral lesion was identified and marked for targeted biopsy based on the PI-RADS v2.1 classification [8]. Prostate lesions with a PI-RADS score of 3 or higher were evaluated as suspicious of PCa and underwent a targeted biopsy. Using the commercially available navigation system Urostation MRI/ultrasound fusion device (Koelis, Meylan, France), the targeted biopsy was performed by one urologist with the previously identified mpMRI lesions superimposed using the T2WI sequence on the real-time TRUS images. Each tumoral lesion was sampled both in the axial and sagittal planes by an end-fire TRUS probe. For the standard biopsy, 12 cores were collected in an extended-sextant template of biopsies from both sides’ lateral and medial aspects of the base, mid, and apical prostate. 

### 2.4. Imaging–Pathology Correlation

A dedicated genitourinary pathologist reviewed the biopsy samples (target and systematic) for each patient. A GS was assigned for each tumor outlined on hematoxylin-eosin (H&E)-stained histology slides using the classification system from the International Society of Urological Pathology (ISUP) [5]. GS ≥ 7 was considered to indicate csPCa. The dedicated clinical radiologist ensured the same target lesions were analyzed at imaging and pathology. The ground truth was provided by histopathological data combined with TRUS-target and systematic biopsies. Our choice of biopsy population is widely validated by the scientific community as it allows for an adequate reference standard for this radiomic study [22].

### 2.5. Segmentation and Radiomic Feature Extraction

For all 86 patients included in the study, a single (index) pathologically confirmed PCa lesion per patient was segmented and analyzed. An experienced radiologist (with 10 years of body MRI experience) and a radiology resident (with 3 years of experience in radiologic reading) participated in the tumor localization. They manually segmented the regions of interest (ROIs) along the tumor border on each section of the ADC map to cover the entire lesion using specialized tools of the Slicer3D v4.10.2 software. Similarly, the ROIs of axial T2WI were drawn with reference to the ADC map. Observers also measured the prostate gland volume using the prolate ellipsoid formula, multiplying the largest anteroposterior (H), transverse (W), and cephalocaudal (L) prostate diameters by 0.524 (H × W × L × π/6). As all images were acquired with the same MRI protocol and resolution, no preprocessing was applied before extracting quantitative image information. Slicer3D v4.10.2 and Python v3.7 were used to extract 2D data from the most representative slice of the previously segmented ROIs of T2WI and ADC maps. The open-source Python Pyradiomics library v2.2.0 was used to compute 97 features from each ROI from ADC and T2 images, excluding normalized features. These were separated into different groups: 2D Shape (*n* = 9), First Order (*n* = 18), and texture features including GLCM (*n* = 24), GLRML (*n* = 14), GLSZM (*n* = 14), GLDM (*n* = 13), and NGTDM (*n* = 5), based on the pixel’s grey level.

### 2.6. Statistical and ML Model Analysis

A feature analysis was performed based on individual performance and correlation, using the nonparametric Mann–Whitney U-test (MWU) statistical hypothesis test [23], Pearson correlation (r < 0.8), Spearman Rank Correlation Coefficient (ρ) with GS classification, and predictive power analysis with bootstrap AUC resulting from the MWU [24,25]. To extract the optimal cutoff value for the prediction of csPCa, a receiver operating characteristic (ROC) curve evaluation was performed. The optimal value corresponded to the maximum Youden Index obtained for the ROC curve [26]. The variables considered for the evaluation were PSA, PSA-D, prostate volume (PV MR), PIRADS-V2.1, and the 194 extracted radiomic features from T2WI and ADC maps. 

The dataset was stratified and split into train/test (80%) and validation (20%) sets. Univariate and multivariate ML models were built for the training/test set using different classifiers, including Logistic Regression (LR), Support Vector Machine (SVM), Naive Bayes (NB), and Classification and Regression Trees (CART). To prevent data leakage and mitigate the impact of data skewness or outliers [27], we scaled the features from the training set using the StandardScaler function and extracted the transformation parameters. This transformation was then applied to the validation/holdout set. The selection of features for the multivariate models was performed automatically, using the Recursive Feature Elimination algorithm (RFE) [27], with LR as the main estimator, allowing a maximum of 10 features. To evaluate the performance of the models, a 2-fold Cross-Validation (CV) was employed for the train/test groups. We used the Repeated Stratified KFold CV technique with 1000 repetitions. The area under the ROC curve (AUC) was calculated to evaluate the classification’s performance. We assessed the performance in a range of 2 to 10 features. The best model and classifier selection considered the mean AUC and its standard deviation.

In this study, we assessed six models—two univariate and four multivariate—for PCa classification (differentiating GS = 6 from GS ≥ 7): PI-RADS v2.1; PSA-D; the best model including quantitative clinical data alone (CL); the best T2WI radiomic model; the best ADC radiomic model; and ultimately, the best combined model, including all available data (T2WI + ADC + CL) as input for the automatic selection algorithm. The performance of the ML models was compared using Frequentist and Bayesian correlated t-tests to obtain the statistical significance of the model differences and guarantee that the results are statistically valid, with a difference of more than 0.01, as suggested in Benavoli et al. [28]. ROC curves were calculated using the best classifier for the best combined model (T2WI + ADC + CL), the PI-RADS v2.1, and PSA-D univariate models. 

**Figure 2:**
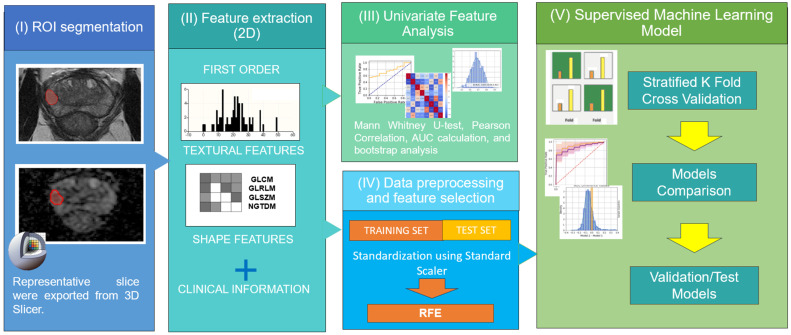
Pipeline encompassing MRI feature analysis and radiomic Machine Learning classification models for patients with prostate cancer (PCa).

The model construction and subsequent statistical analyses detailed above were executed utilizing Python version 3.7. The Scikit-learn package was used to build, train, and validate the ML classification models. For the statistical analysis, the Matplotlib package provided the required tests. Statistical significance was determined at a threshold of *p*-value < 0.05. A visual representation of this workflow is summarized in Figure 2.

## 3. Results

### 3.1. Patient Population 

Histopathological findings revealed a total of 86 prostate cancer lesions. Within this cohort, 20 lesions (23%) exhibited a GS score of 6, 28 lesions (33%) were classified as 3 + 4, 21 lesions (24%) were categorized as 4 + 3, and 17 lesions (20%) had a GS score of ≥8. For GS classification, the low-risk group (GS = 6) considered 20 patients, and the high-risk group (GS ≥ 7) included 66 patients. An overview of the clinical indicators for the study participants is presented in Table 2.

### 3.2. Univariate Performance 

We selected the features for the univariate analysis based on their statistical significance and AUC performance for the classification task, discarding highly correlated features with lower performance. The evaluation included the 97 radiomic features extracted from T2WI and ADC images and the additional clinical and image information. Table 3 shows the results for the three most statistically significant values obtained in each dataset category. This table only considers features that were not highly correlated (r > 0.8). 

In our study, the best individual feature for classifying csPCa and non-csPCa was PSA-D (AUC = 0.77), with a cutoff value for csPCa of 0.14 ng/mL^2^. This feature also exhibited the highest correlation rank value with GS (ρ = 0.46) among all the categories. However, the variance (95% C.I. [0.66–0.87]) was in the same range as the rest of the selected features. Even though PI-RADS v2.1 score (AUC = 0.66 [0.57–0.74], ρ = 0.28) did not make it to the highest three AUC values from its category, the results are similar to those found for PSA (AUC = 0.66 [0.52–0.78], ρ = 0.37). 

From the radiomic features, the ADC maps provided information that allowed two features to reach a maximum AUC value of 0.75, being the highest value after PSA-D. The one with the smaller C.I. corresponded to a first-order type feature (Minimum) of the ADC map and showed a negative correlation with GS. The Spearman correlations between GS and ADC features generally had better values than those obtained for the T2WI image features.

### 3.3. Evaluation of ML Multivariate Models

The resulting set sizes from the data split step were 68 patients for the training set and 18 patients for the holdout set. The best results for each classification can be found in Table 4, with the best classifier for most models being LR (6 out of 8). The best model had a mean AUC of 0.91 [0.76–0.99] for differentiating non-csPCa (GS = 6) vs. csPCa (GS ≥ 7), with a total of 10 selected features and a validation/holdout AUC of 0.80. The chosen features for each classification are presented in Table 5.

The comparison between the best multivariate combined (T2WI + ADC + CL) model and the best model based only on quantitative clinical information (CL: PSA-D, PV MR, and PSA) yielded a probability for the combined model outperforming the pure clinical model of 0.96 for the training set.

### 3.4. Combined ML Models vs. PI-RADS vs. PSA-D 

**Figure 3:**
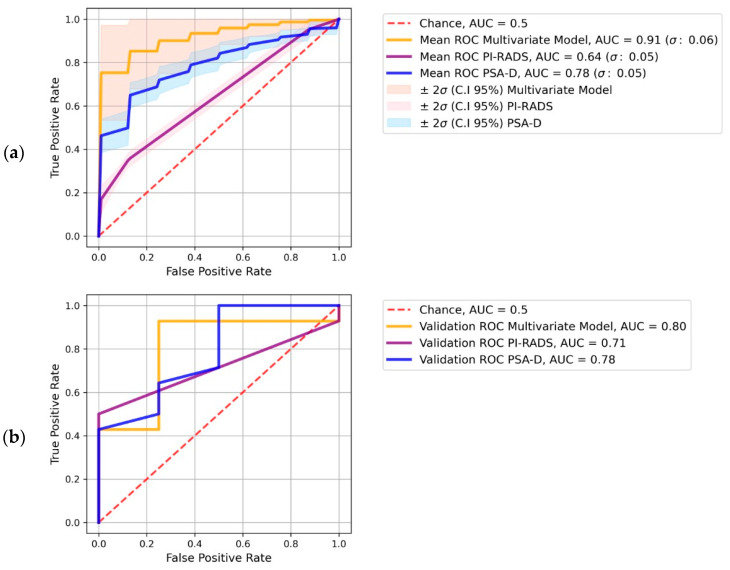
ROC curves for predicting PCa aggressiveness (GS = 6 vs. GS ≥ 7), including AUC scores for the best multivariate model and for univariate PI-RADS and PSA-D model. (**a**) ROC curves with 95% confidence intervals for the training dataset (repeated 2-fold CV). (**b**) ROC curves and AUC scores for the corresponding validation dataset.

The training set ROC curves for each model are presented in Figure 3. The validation AUC of PI-RADS-v2.1 was 0.71 (training AUC score: 0.64 [0.55–0.73]), while for PSA-D, it was 0.78 (training AUC score: 0.79 [0.67–0.90]), both using LR as the best classifier. 

According to the Bayesian analysis, our multivariate model performs better than PI-RADS v2.1 and PSA-D. For PI-RADS v2.1, the probability value for the multivariate model exceeding the performance among the training AUC values was 0.99. For PSA-D, the Bayesian comparison yielded a probability value of 0.93. These results demonstrate statistical significance in favor of the multivariate model’s superior performance compared with the univariate clinical models for the binary classification task within this cohort.

## 4. Discussion

This study presents an ML-based framework based on biparametric MRI-derived T2WI and ADC radiomic features, combined with clinical information, to identify the best-performing model to classify csPCa. Our study’s key finding was that MRI-derived radiomics (T2WI/ADC) features exhibit a good performance as quantitative imaging biomarkers for PCa classification in a cohort of Hispanic patients, a population where data are scarce and only a few studies have been published [29,30]. Our combined multivariate ML model using radiomic and clinical variables outperforms the classically used qualitative PI-RADS v2.1 and clinical indicators like PSA-D for the classification of clinically significant PCa.

Our findings agree with previous studies assessing the role of texture feature analysis using T2WI and ADC. Wibmer et al. [21] showed that several Haralick-based texture features (entropy, energy, correlation, inertia, and homogeneity) extracted from T2WI/ADC images could be helpful for Pca detection and that ADC texture parameters correlate with tumor aggressiveness. Among the selected features used in our best multivariate model for the classification of csPCa (GS ≥ 7), we obtained maximum ADC, which had a moderate individual performance and a negative correlation with GS. A similar inclusion of maximum ADC within a combined multivariate model was also noted by Wóznicki et al. [31], albeit in the context of a volume of interest (VOI). Perhaps, the good correlation (≥80%) of this parameter with mean ADC, which has been studied as an imaging classification biomarker previously by Donati O. F. et al. [12], could help to interpret the rationale behind this feature selection. 

Our study corroborates and expands upon earlier research that affirms the superiority of a multivariate ML model using radiomic and clinical variables over PI-RADS classification and serum markers such as PSA-D. Woźnicki et al. [31], in a model combining T2WI/ADC radiomic and clinical data (PSA-D, digital rectal examination (DRE)), achieved higher predictive AUC for differentiation of csPCa vs. non-csPCa in the test cohort when compared with PI-RADS and mean ADC (AUCs of 0.84, 0.68 and 0.57 respectively)—consistent with our findings. Moreover, Varghese et al. also established the enhanced performance of ML-based radiomic analysis over PI-RADS evaluation [32]. Similar to our methodology, they used a 2D radiomic approach on T2WI/ADC features to classify csPCa and demonstrated a robust performance by validating the model with an external cohort. 

In contrast to our findings, Bonekamp et al. [33] observed similar performance between an ML-based radiomic approach and the quantitative assessment of mean ADC without the added value of incorporating T2WI MRI-derived texture features within their specific cohort. In a separate study, Gresser et al. [34] explored the potential of an MRI-derived radiomic model using T2WI/ADC data on PI-RADS ≥ 3 prostate lesions using a real-world dataset. Their analysis unveiled mean AUCs ranging from 0.78 to 0.83 for distinguishing between csPCa and non-csPCa. Although these values were generally higher than PI-RADS, mean ADC, or PSA-D, the differences lacked statistical significance. They concluded that the limited clinical utility of this approach stems from its susceptibility to low robustness and high result variability.

Our combined model attained an AUC of 0.80 in the validation dataset, effectively distinguishing between csPCa and indolent non-csPCa cases. These outcomes are consistent with recent systematic reviews conducted by Castillo et al. [35] and Sunchentev et al. [36], where reported AUCs ranged from 0.75 to 0.88. These studies employed semiautomated artificial intelligence methods for detecting csPCa, incorporating both peripheral and transition zone lesions. The existing body of evidence reinforces our results, especially within the unique demographic context of our Hispanic patient population.

PSA-D and PV were the clinical parameters included in the model. Since PSA production is deregulated in PCa, PSA-D accounts for this disproportionate rise in prostate volume. In our study, PSA-D exhibited a cutoff value of 0.14 ng/mL^2^, which lies within the range of 0.10–0.15 ng/mL^2^, highlighted by Bruno et al. [37] as indicative of clinically significant PCa suspicion. PV was also included, contributing positively to its final performance. Its negative Spearman correlation is probably explained by the fact that smaller prostates can concentrate a more significant amount of cancer by excluding pathologies such as benign prostatic hyperplasia, which has been seen to be a protective factor in these cases, as seen in Yamashiro et al.’s systematic review [38]. Our model uses PSA-D to predict more aggressive tumor histology, which affirms that serum and imaging biomarkers can be synergistic. 

This study developed a systematic ML-based framework to acknowledge the critical role of feature and classifier selection in radiomic model performance. Its objective was to establish an objective and reliable risk classifier by initially addressing the reduction in feature space dimensionality. RFE enabled us to identify the most informative radiomic and clinical features from the dataset and select the optimal number for each multivariate model within our limit of 10. This tool has been previously used to reduce dimensionality in MR-based PCa risk classification by other authors [39]. We also compared different Machine Learning classifiers (LR, SVM, NB, and CART) for building the radiomic models. As previously reported, LR was the best and most common classifier selected for the distinction of csPCa [35,39]. This methodology, coupled with Bayesian analysis, extends the comprehension of model significance beyond *p*-values, facilitating a more insightful interpretation of statistical outcomes. 

Respecting the validation process, our results present some cases that escaped 95% AUC C.I. To explain these results, we evaluated the distribution of values in our patient cohort, finding outlier features (test data values out of 2σ range) with respect to the training cohort. Moreover, the imbalanced nature of our dataset regarding the distribution of patients in each GS group could have influenced this behavior. In future works, different metrics to the AUC for selecting features and models could provide more stable results, as suggested by Jeni L. et al. [40].

Despite extensive recent evidence supporting the potential use of radiomics in PCa detection, aggressiveness assessment, and treatment decision-making assistance, several studies have highlighted the substantial heterogeneity and poor reproducibility of the developed predictive models [19,36]. As a result, various initiatives have acknowledged the need for radiomics standardization to increase repeatability and bring radiomics research into clinical practice [41]. A recent work by Castaldo et al. [42] commented on the importance of using IBSI-compliant software (such as the open-source Python Pyradiomics library) and standardizing radiomic features as part of the pipeline strategies that might improve the reproducibility of these potential noninvasive biomarkers for PCa assessment, both of which were used in our study.

Our results have important clinical implications, since new diagnostic tools for PCa imaging studies are emerging, helping clinicians to differentiate nonsignificant vs. clinically significant PCa, avoiding overtreatment, which may lead to potential surgical complications and deterioration in life quality. Our findings support the concept of an MRI-guided therapy route and highlight the possibility of novel imaging-based radiomics and clinical biomarkers for risk classification in patients with PCa. Automatic ML assessment of the prostate gland and characterization of suspicious index lesions in MRI could provide more comprehensive imaging biomarkers for improved cancer identification and risk stratification.

### Limitations

We acknowledge several limitations of our study. First, because of the retrospective nature, there might have been a potential selection bias in the patient sample. Also, the MRI examination was carried out as part of the diagnostic process, and a texture/radiomic analysis was retrospectively applied to a confirmed cancerogenic lesion using radiomics for lesion characterization rather than lesion detection. Second, we use MRI-targeted biopsy specimens as the ground truth instead of radical prostatectomy, which offers the definitive assessment of PCa lesions; however, our target biopsy population allows a correct imaging–pathology correlation and limits the overrepresentation of intermediate-risk PCa disease [36]. We decided to use an analysis of index lesions only to avoid statistical clustering, because these lesions usually drive management strategies and patient outcomes [43]. Third, we used a non-open-source single-center database, with all MRI studies performed by the same vendor/machine and without an external testing validation set, which limits the reproducibility and clinical applicability of the proposed predictive models. In future works, we will explore our developed algorithm in different medical centers as part of a multicenter study, with recently proposed frameworks that facilitate the harmonization of multicenter radiomic features to develop robust biomarkers in PCa clinical practice [42].

Fourth, even though our work used a moderately large sample compared with other studies, the number of cases included is still a limitation in this ML/radiomics study, and bigger series are needed. Finally, a subjective operator-based ROI segmentation of target lesions by two observers in consensus was used in our research, resulting in a potential ROI placement bias and limiting the generalizability of the developed predictive model. New AI-based, fully automated methods for lesion segmentation might be explored in the future to help overcome this problem, as well as 3D volumetric segmentation. However, our results agree with previous studies while following an adequate methodology, as stated by Stanzione et al. [18].

## 5. Conclusions

Radiomics, in combination with ML tools, is a potentially valuable tool for objective PCa aggressiveness imaging classification. Although further prospective large-scale cohorts with external validation studies are needed, our results suggest that a combined multivariate ML model using radiomic (T2WI/ADC) and clinical variables has a potential role in predicting csPCa, particularly within the unique context of our Hispanic patient population, which could help to direct patient management. In this work, our multivariate ML model outperforms the classically used qualitative PI-RADS v2.1 and clinical indicators like PSA-D for the classification of csPCa. Future work on automatic segmentation, data preprocessing, augmentation techniques, and protocolization is necessary to provide greater reproducibility to this promising diagnostic tool.

## Figures and Tables

**Table 1:** Sequence parameters for T2WI and DWI sequences.

Image Type	Acquisition Parameters	
T2WI	Matrix (pixels)	200 × 190
	TE (ms)	110
	TR (ms)	3200
	Slice thickness (mm)	3
DWI	Matrix (pixels)	84 × 67
	TE (ms)	98
	TR (ms)	7100
	Slice thickness (mm)	3
	Diffusion gradient	b100–500–1000–2000

Scanner Philips Ingenia, magnetic field strength of 3 T. TE = echo time; TR = repetition time; DWI = diffusion-weighted image.

**Table 2:** Patient demographics and clinical characteristics.

Patient Characteristics	Data
Total number of patients with PCa included	86
Age (years), mean ± σ	66.10 ± 8.13
Comorbidities *n* (%)	63 (73)
Arterial hypertension	38 (44)
Diabetes Mellitus	19 (22)
Tobacco	13 (15)
PSA (ng/mL), median (IQR)	7.4 (10.6–4.7)
Prostate volume (ml), median (IQR)	43.5 (58–33)
Lesion size (mm), median (IQR)	10 (15.8–8.0)
PSA-D MR (ng/mL^2^), median (IQR)	0.15 (0.26–0.10)
Localization *n* (%)	
Transition Zone (TZ)	5 (6)
Peripherical Zone (PZ)	81 (94)
PI-RADS *n* (%)	
3	2 (2)
4	60 (70)
5	24 (28)
Gleason Score *n* (%)	
6	20 (23)
7 (3 + 4)	28 (33)
7 (4 + 3)	21 (33)
8	5 (6)
9	12 (14)

σ = standard deviation; PSA = prostate-specific antigen; IQR = interquartile range; PCa = prostate cancer: PSA-D MR = prostate-specific antigen density by magnetic resonance.

**Table 3:** AUC Scores of the three best radiomic and clinical features for each category.

Type	Texture/Clinical Parameter	AUC (95% C.I.)	Cutoff	C-GS (𝜌)
T2WI	glszm-*GrayLevelNonUniformity*	0.72 (0.59–0.83)	5.6	0.36
	shape-*Elongation*	0.70 (0.55–0.82)	0.74	−0.18
	ngtdm-*Coarseness*	0.70 (0.59–0.81)	0.02	−0.31
ADC	firstorder-Minimum	0.75 (0.64–0.85)	316 × 10^−6^ mm^2^/s	−0.36
	gldm-*DependenceVariance*	0.75 (0.61–0.86)	0.10	0.37
	gldm-*GrayLevelNonUniformity*	0.73 (0.59–0.84)	1.84	0.44
Clinical	PSA-D	0.77 (0.66–0.87)	0.14 ng/mL^2^	0.46
	PV MR	0.69 (0.55–0.81)	45 mL	−0.24
	PSA	0.66 (0.52–0.78)	8 ng/ml	0.37

Cutoff (threshold) and C-GS (Spearman correlation rank). For highly correlated features (r > 0.8), we kept the one exhibiting the best AUC. PSA = prostate-specific antigen; PSA-D = prostate-specific-antigen density; PV MRI = prostate volume measured by MRI.

**Table 4:** Performance of the ML models for the non-csPCa (GS = 6) vs csPCa (G ≥ 7) classification task.

Dataset	*n*°	Best Classifier	AUC (σ) [95% C.I.]	AUC_val_	acc_val_
T2WI	4	LR	0.85 (0.05) [0.71–0.94]	0.55	0.67
ADC	6	LR	0.81 (0.08) [0.56–0.94]	0.71	0.88
CL	3	LR	0.76 (0.06) [0.62–0.87]	0.80	0.83
T2WI + ADC + CL	10	LR	0.91 (0.06) [0.76–0.99]	0.80	0.83

*n*° refers to the number of selected features with the best train/test performance in the dataset. The last two columns correspond to the validation (holdout) results. CL = quantitative clinical information; σ = Standard deviation; C.I. = Confidence Interval; LR = Logistic Regression; SVM = Support Vector Machine; NB = Naive Bayes; AUC_val_ = validation AUC; acc_val_ = validation accuracy.

**Table 5:** Selected features by RFE using Logistic Regression (LR) as an estimator for the best ML multivariate model, i.e., dataset T2WI + ADC + CL.

Information Type	GS = 6 vs. GS ≥ 7
T2WI	glcm-*Imc1*
	glrlm-*LongRunLowGrayLevelEmphasis*
	glrlm-*ShortRunHighGrayLevelEmphasis*
	glrlm-*RunLengthNonUniformity*
ADC	firstorder-Maximum
	ngtdm-*Busyness*
	glcm-*DifferenceVariance*
	glrlm-*ShortRunEmphasis*
Clinical	PSA-D
	PV MR

GS = Gleason Score; PSA-D = prostate-specific antigen density; PV MRI = prostate volume measured by MRI.

## Data Availability

The data presented in this study might be available upon request from the corresponding author. The data are not publicly available due to privacy.

## References

[1] Maddams J., Utley M., Møller H. (2012). Projections of cancer prevalence in the United Kingdom 2010–2040. Br. J. Cancer.

[2] Aihara M., Wheeler T.M., Ohori M., Scardino P.T. (1994). Heterogeneity of prostate cancer in radical prostatectomy specimens. Urology.

[3] Epstein J.I., Egevad L., Amin M.B., Delahunt B., Srigley J.R., Humphrey P.A., Grading Committee (2016). The 2014 International Society of Urological Pathology (ISUP) Consensus Conference on Gleason Grading of Prostatic Carcinoma: Definition of Grading Patterns and Proposal for a New Grading System. Am. J. Surg. Pathol..

[4] D’Amico A.V., Moul J., Carroll P.R., Sun L., Lubeck D., Chen M.H. (2003). Cancer-specific mortality after surgery or radiation for patients with clinically localized prostate cancer managed during the prostate-specific antigen era. J. Clin. Oncol. Off. J. Am. Soc. Clin. Oncol..

[5] Ahmed H.U., El-Shater Bosaily A., Brown L.C., Gabe R., Kaplan R., Parmar M.K., Collaco-Moraes Y., Ward K., Hindley R.G., Freeman A. (2017). PROMIS study group Diagnostic accuracy of multi-parametric MRI and TRUS biopsy in prostate cancer (PROMIS): A paired validating confirmatory study. Lancet.

[6] Kasivisvanathan V., Rannikko A.S., Borghi M., Panebianco V., Mynderse L.A., Vaarala M.H., Briganti A., Budäus L., Hellawell G., Hindley R.G. (2018). PRECISION Study Group Collaborators MRI-Targeted or Standard Biopsy for Prostate-Cancer Diagnosis. N. Engl. J. Med..

[7] Rouvière O., Puech P., Renard-Penna R., Claudon M., Roy C., Mège-Lechevallier F., Decaussin-Petrucci M., Dubreuil-Chambardel M., Magaud L., Remontet L. (2019). MRI-FIRST Investigators Use of prostate systematic and targeted biopsy on the basis of multiparametric MRI in biopsy-naive patients (MRI-FIRST): A prospective, multicentre, paired diagnostic study. Lancet Oncol..

[8] Turkbey B., Rosenkrantz A.B., Haider M.A., Padhani A.R., Villeirs G., Macura K.J., Tempany C.M., Choyke P.L., Cornud F., Margolis D.J. (2019). Prostate Imaging Reporting and Data System Version 2.1: 2019 Update of Prostate Imaging Reporting and Data System Version 2. Eur. Urol..

[9] Park S.Y., Jung D.C., Oh Y.T., Cho N.H., Choi Y.D., Rha K.H., Hong S.J., Han K. (2016). Prostate Cancer: PI-RADS Version 2 Helps Preoperatively Predict Clinically Significant Cancers. Radiology.

[10] Park K.J., Choi S.H., Lee J.S., Kim J.K., Kim M.H. (2020). Interreader Agreement with Prostate Imaging Reporting and Data System Version 2 for Prostate Cancer Detection: A Systematic Review and Meta-Analysis. J. Urol..

[11] Schieda N., Lim C.S., Zabihollahy F., Abreu-Gomez J., Krishna S., Woo S., Melkus G., Ukwatta E., Turkbey B. (2021). Quantitative Prostate MRI. J. Magn. Reson. Imaging.

[12] Donati O.F., Mazaheri Y., Afaq A., Vargas H.A., Zheng J., Moskowitz C.S., Hricak H., Akin O. (2014). Prostate cancer aggressiveness: Assessment with whole-lesion histogram analysis of the apparent diffusion coefficient. Radiology.

[13] Jung S.I., Donati O.F., Vargas H.A., Goldman D., Hricak H., Akin O. (2013). Transition zone prostate cancer: Incremental value of diffusion-weighted endorectal MR imaging in tumor detection and assessment of aggressiveness. Radiology.

[14] Rosenkrantz A.B., Neil J., Kong X., Melamed J., Babb J.S., Taneja S.S., Taouli B. (2010). Prostate cancer: Comparison of 3D T2-weighted with conventional 2D T2-weighted imaging for image quality and tumor detection. AJR Am. J. Roentgenol..

[15] Surov A., Meyer H.J., Wienke A. (2020). Correlations between Apparent Diffusion Coefficient and Gleason Score in Prostate Cancer: A Systematic Review. Eur. Urol. Oncol..

[16] Smith C.P., Czarniecki M., Mehralivand S., Stoyanova R., Choyke P.L., Harmon S., Turkbey B. (2019). Radiomics and radiogenomics of prostate cancer. Abdom. Radiol..

[17] Penzkofer T., Padhani A.R., Turkbey B., Haider M.A., Huisman H., Walz J., Salomon G., Schoots I.G., Richenberg J., Villeirs G. (2021). ESUR/ESUI position paper: Developing artificial intelligence for precision diagnosis of prostate cancer using magnetic resonance imaging. Eur. Radiol..

[18] Stanzione A., Gambardella M., Cuocolo R., Ponsiglione A., Romeo V., Imbriaco M. (2020). Prostate MRI radiomics: A systematic review and radiomic quality score assessment. Eur. J. Radiol..

[19] Cuocolo R., Cipullo M.B., Stanzione A., Romeo V., Green R., Cantoni V., Ponsiglione A., Ugga L., Imbriaco M. (2020). Machine learning for the identification of clinically significant prostate cancer on MRI: A meta-analysis. Eur. Radiol..

[20] Nketiah G., Elschot M., Kim E., Teruel J.R., Scheenen T.W., Bathen T.F., Selnæs K.M. (2017). T2-weighted MRI-derived textural features reflect prostate cancer aggressiveness: Preliminary results. Eur. Radiol..

[21] Wibmer A., Hricak H., Gondo T., Matsumoto K., Veeraraghavan H., Fehr D., Zheng J., Goldman D., Moskowitz C., Fine S.W. (2015). Haralick texture analysis of prostate MRI: Utility for differentiating non-cancerous prostate from prostate cancer and differentiating prostate cancers with different Gleason scores. Eur. Radiol..

[22] Siddiqui M.M., Rais-Bahrami S., Truong H., Stamatakis L., Vourganti S., Nix J., Hoang A.N., Walton-Diaz A., Shuch B., Weintraub M. (2013). Magnetic resonance imaging/ultrasound-fusion biopsy significantly upgrades prostate cancer versus systematic 12-core transrectal ultrasound biopsy. Eur. Urol..

[23] Corder G., Foreman D. (2010). Nonparametric Statistics for Non-Statisticians: A Step-by-Step Approach. Int. Stat. Rev..

[24] Mason S.J., Graham N.E. (2002). Areas beneath the relative operating characteristics (ROC) and relative operating levels (ROL) curves: Statistical significance and interpretation. Q. J. R. Meteorol. Soc..

[25] Davison A., Hinkley D. (1997). Confidence Intervals. Bootstrap Methods and Their Application.

[26] Youden W.J. (1950). Index for rating diagnostic tests. Cancer.

[27] Pedregosa F., Varoquaux G., Gramfort A., Michel V., Thirion B., Grisel O., Blondel M., Prettenhofer P., Weiss R., Dubourg V. (2011). Scikit-learn: Machine Learning in Python. J. Mach. Learn. Res..

[28] Benavoli A., Corani G., Demšar J., Zaffalon M. (2017). Time for a change: A tutorial for comparing multiple classifiers through Bayesian analysis. J. Mach. Learn. Res..

[29] Saltzman R.G., Zucker I., Campbell K., Gandhi D.A., Otiono K., Weber A., Masterson T.A., Ramasamy R. (2022). An evaluation of race-based representation among men participating in clinical trials for prostate cancer and erectile dysfunction. Contemp. Clin. Trials Commun..

[30] Javier-DesLoges J., Nelson T.J., Murphy J.D., McKay R.R., Pan E., Parsons J.K., Kane C.J., Kader A.K., Derweesh I.H., Nodora J. (2022). Disparities and trends in the participation of minorities, women, and the elderly in breast, colorectal, lung, and prostate cancer clinical trials. Cancer.

[31] Woźnicki P., Westhoff N., Huber T., Riffel P., Froelich M.F., Gresser E., von Hardenberg J., Mühlberg A., Michel M.S., Schoenberg S.O. (2020). Multiparametric MRI for Prostate Cancer Characterization: Combined Use of Radiomics Model with PI-RADS and Clinical Parameters. Cancers.

[32] Varghese B., Chen F., Hwang D., Palmer S.L., De Castro Abreu A.L., Ukimura O., Aron M., Aron M., Gill I., Duddalwar V. (2019). Objective risk stratification of prostate cancer using machine learning and radiomics applied to multiparametric magnetic resonance images. Sci. Rep..

[33] Bonekamp D., Kohl S., Wiesenfarth M., Schelb P., Radtke J.P., Götz M., Kickingereder P., Yaqubi K., Hitthaler B., Gählert N. (2018). Radiomic Machine Learning for Characterization of Prostate Lesions with MRI: Comparison to ADC Values. Radiology.

[34] Gresser E., Schachtner B., Stüber A.T., Solyanik O., Schreier A., Huber T., Froelich M.F., Magistro G., Kretschmer A., Stief C. (2022). Performance variability of radiomics machine learning models for the detection of clinically significant prostate cancer in heterogeneous MRI datasets. Quant. Imaging Med. Surg..

[35] Castillo T J.M., Arif M., Niessen W.J., Schoots I.G., Veenland J.F. (2020). Automated Classification of Significant Prostate Cancer on MRI: A Systematic Review on the Performance of Machine Learning Applications. Cancers.

[36] Sushentsev N., Moreira Da Silva N., Yeung M., Barrett T., Sala E., Roberts M., Rundo L. (2022). Comparative performance of fully-automated and semi-automated artificial intelligence methods for the detection of clinically significant prostate cancer on MRI: A systematic review. Insights Imaging.

[37] Bruno S.M., Falagario U.G., d’Altilia N., Recchia M., Mancini V., Selvaggio O., Sanguedolce F., Del Giudice F., Maggi M., Ferro M. (2021). PSA Density Help to Identify Patients with Elevated PSA Due to Prostate Cancer Rather Than Intraprostatic Inflammation: A Prospective Single Center Study. Front. Oncol..

[38] Yamashiro J.R., de Riese W.T. (2021). Any Correlation Between Prostate Volume and Incidence of Prostate Cancer: A Review of Reported Data for the Last Thirty Years. Res. Rep. Urol..

[39] Zhou Y., Yuan J., Xue C., Poon D.M.C., Yang B., Yu S.K., Cheung K.Y. (2023). A pilot study of MRI radiomics for high-risk prostate cancer stratification in 1.5 T MR-guided radiotherapy. Magn. Reson. Med..

[40] Jeni L.A., Cohn J.F., De La Torre F. Facing Imbalanced Data Recommendations for the Use of Performance Metrics. Proceedings of the 2013 Humaine Association Conference on Affective Computing and Intelligent Interaction.

[41] Da-Ano R., Lucia F., Masson I., Abgral R., Alfieri J., Rousseau C., Mervoyer A., Reinhold C., Pradier O., Schick U. (2021). A transfer learning approach to facilitate ComBat-based harmonization of multicentre radiomic features in new datasets. PLoS ONE.

[42] Castaldo R., Brancato V., Cavaliere C., Trama F., Illiano E., Costantini E., Ragozzino A., Salvatore M., Nicolai E., Franzese M. (2022). A Framework of Analysis to Facilitate the Harmonization of Multicenter Radiomic Features in Prostate Cancer. J. Clin. Med..

[43] Stamey T.A., McNeal J.M., Wise A.M., Clayton J.L. (2001). Secondary cancers in the prostate do not determine PSA biochemical failure in untreated men undergoing radical retropubic prostatectomy. Eur. Urol..

